# Methyl jasmonate maintains postharvest quality of *Gastrodia elata* by coordinately regulating ROS homeostasis, phenylpropanoid biosynthesis, and energy metabolism

**DOI:** 10.3389/fnut.2026.1873026

**Published:** 2026-07-29

**Authors:** Qiong Wang, Yaru Zhao, Yun Zhou, Zuxin He, Na Li, Yani Zhang, Xiaorong Ren, Zhen Zhang

**Affiliations:** 1 Pingliang Products Quality Supervision and Inspection Center, Pingliang, Gansu, China; 2 Pingliang Agricultural Products Quality and Safety & Inspection and Testing Center, Pingliang, Gansu, China; 3 Qingyang Food Inspection and Testing Center, Qingyang, Gansu, China; 4 College of Food Science and Engineering, Gansu Agricultural University, Lanzhou, Gansu, China

**Keywords:** antioxidant capacity, energy metabolism, *Gastrodia elata*, methyl jasmonate, phenylpropanoid metabolism

## Abstract

*Gastrodia elata* is an economically important medicinal and edible plant, but its fresh tubers are highly susceptible to postharvest decay and quality deterioration during storage. Methyl jasmonate (MeJA) is well established as an effective postharvest treatment for preserving the quality of horticultural produce. In this investigation, we evaluated the potential of MeJA treatment to improve the postharvest quality of *G. elata* during storage. Our findings indicated that MeJA markedly lowered decay incidence and inhibited the elevation of respiration rate and malondialdehyde (MDA) accumulation. MeJA also decreased superoxide anion generation (O_2_•−) and hydrogen peroxide (H_2_O_2_) levels, while stimulating the activities and transcription levels of superoxide dismutase (SOD), catalase (CAT), peroxidase (POD), ascorbate peroxidase (APX), glutathione reductase (GR), monodehydroascorbate reductase (MDHAR), and dehydroascorbate reductase (DHAR). In parallel, the levels of the non-enzymatic antioxidants ascorbic acid (AsA) and glutathione (GSH) were elevated. Moreover, MeJA treatment elevated the contents of total phenolics, flavonoids, and lignin in *G. elata*, accompanied by increased activities and gene expression of major phenylpropanoid-pathway enzymes, including phenylalanine ammonia-lyase (PAL), cinnamate 4-hydroxylase (C4H), 4-coumarate ligase (4CL), and cinnamyl alcohol dehydrogenase (CAD). MeJA further enhanced the activities and transcription levels of H^+^-ATPase, succinate dehydrogenase (SDH), Ca^2+^-ATPase, and cytochrome c oxidase (CCO), thereby helping maintain cellular energy homeostasis in *G. elata*. In addition, MeJA upregulated the transcription of the regulatory factors *GeMYB86, GeNAC22*, and *GeWRKY35/70*. Overall, these findings suggest that MeJA preserves the postharvest quality of *G. elata* through coordinated regulation of reactive oxygen species metabolism, phenylpropanoid biosynthesis, and energy metabolism, which in turn reinforces antioxidant defense and reduces oxidative injury.

## 1 Introduction

*Gastrodia elata* is an orchid species of the genus *Gastrodia*, and its tubers are valued for both edible and medicinal purposes, representing a typical food–medicine homologous resource (1). In recent years, increasing attention to the medicinal properties and nutritional benefits of fresh *G. elata* has driven a steady rise in market demand. Nevertheless, the tubers of fresh *G. elata* have a high water content and poor storage tolerance, making them highly susceptible to spoilage during storage and distribution, with rot losses reaching 15–30%, which severely limits the high-quality development of the fresh *G. elata* industry (2).

Postharvest physiological regulation and defense signaling in plants are both strongly influenced by reactive oxygen species (ROS). On the one hand, ROS can directly inhibit pathogen growth while also operating as signaling factors that trigger defense-related gene transcription. By contrast, excessive accumulation of ROS may trigger lipid peroxidation, impair cellular membrane integrity, and disrupt normal metabolism, thereby accelerating tissue senescence and postharvest quality deterioration (3, 4). To preserve intracellular ROS homeostasis, plants depend on an antioxidant defense network consisting of superoxide dismutase (SOD), catalase (CAT), peroxidase (POD), ascorbate peroxidase (APX), and glutathione reductase (GR), which function in a coordinated manner to eliminate excessive ROS (5). It has been reported that a variety of postharvest elicitation treatments can strengthen antioxidant capacity and retard senescence in fruits and vegetables by affecting ROS metabolism, including melatonin (6), nitric oxide (7), γ-aminobutyric acid (5), and methyl jasmonate (MeJA) (8).

In plant secondary metabolism, the phenylpropanoid pathway is crucial for antioxidant protection and defense against disease. Involving phenylalanine ammonia-lyase (PAL), cinnamate 4-hydroxylase (C4H), and 4-coumarate:CoA ligase (4CL) as the main regulatory enzymes, this pathway promotes the biosynthesis and accumulation of phenolic acids, flavonoids, lignin, and other defense-related secondary metabolites (9, 10). These metabolites not only possess strong free radical-scavenging capacity and antioxidant activity, but also reinforce cell wall structure and inhibit pathogen invasion, thereby enhancing the self-protective capacity of plant tissues (11, 12). Available evidence indicates that modulation of the phenylpropanoid pathway after harvest contributes to enhanced disease resistance and antioxidant capacity in a range of horticultural crops. For example, exogenous caffeic acid and epicatechin can increase the contents of total phenolics, flavonoids, and lignin in apple fruit by activating PAL, C4H, and 4CL, thereby enhancing resistance to gray mold (13). Similarly, melatonin treatment can improve postharvest disease resistance in tomato fruit by promoting PAL, 4CL, and POD activities as well as the accumulation of phenolics and lignin (14).

In addition, energy metabolism is vital for maintaining postharvest quality and postponing senescence in horticultural products. An adequate energy supply is essential for preserving membrane integrity, sustaining normal respiratory metabolism, and ensuring effective stress responses, whereas declines in ATP content and imbalances in energy charge are often closely associated with postharvest senescence, physiological disorders, and reduced disease resistance (15, 16). Previous studies have shown that appropriate postharvest treatments can maintain a high energy status by increasing ATP and ADP contents, preserving a higher energy charge, and enhancing the activities of mitochondrial energy metabolism-related enzymes, such as succinate dehydrogenase (SDH), cytochrome c oxidase (CCO), H^+^-ATPase, and Ca^2+^-ATPase, thereby alleviating stress damage and delaying tissue senescence (17–19). Notably, ROS metabolism, phenylpropanoid metabolism, and energy metabolism do not function independently, but rather jointly participate in the regulation of postharvest stress resistance and quality maintenance.

MeJA is a key endogenous signaling molecule in plants and contributes substantially to regulating growth and development, senescence, and stress responses (8, 20). As an effective postharvest elicitor, MeJA has been widely applied in studies on the preservation and disease control of horticultural products. Accumulating evidence suggests that MeJA treatment can delay postharvest senescence and enhance stress resistance by regulating the antioxidant system and mitochondrial energy metabolism (8, 19). Therefore, MeJA shows considerable potential for maintaining postharvest quality and inducing resistance. Compared with conventional chemical preservatives, MeJA has the advantages of functioning as a natural plant signaling molecule and activating endogenous defense responses, and its effectiveness has been demonstrated in various harvested fruits and vegetables, including its roles in reducing decay, maintaining antioxidant capacity, regulating phenylpropanoid metabolism, and delaying quality deterioration (8). Meanwhile, fresh *G. elata* tubers have received increasing attention for fresh consumption and processing because of their edible and medicinal value (1). Recently, Dong et al. (21) reported that melatonin maintained the postharvest quality of fresh *G. elata* tubers by regulating antioxidant ability, phenylpropanoid metabolism, and energy metabolism during storage, indicating that exogenous regulatory substances can effectively modulate postharvest physiological metabolism in fresh *G. elata*. However, melatonin and MeJA differ markedly in their biological functions and regulatory characteristics. Melatonin is generally recognized as an antioxidant-related regulator involved in ROS homeostasis and stress alleviation, whereas MeJA is a jasmonate-derived signaling molecule closely associated with defense induction and stress-responsive metabolic regulation. Therefore, the regulatory effects of melatonin on fresh *G. elata* cannot be directly extrapolated to MeJA. However, compared with common fruits and vegetables, postharvest preservation studies on fresh *G. elata* remain relatively limited, and the potential application of MeJA in maintaining the storage quality of fresh *G. elata* tubers has not been sufficiently clarified. Therefore, investigating the effect of MeJA on fresh *G. elata* is both scientifically meaningful and practically relevant for developing safe preservation strategies for this medicinal and edible tuber. Systematic studies on the dynamic changes in ROS metabolism, phenylpropanoid metabolism, and energy metabolism, as well as their coordinated regulatory mechanisms in *G. elata* after MeJA treatment, are still lacking. Against this background, this study was designed to examine the contribution of MeJA treatment on the quality of *G. elata* during postharvest storage and to further elucidate the synergistic roles of ROS metabolism, phenylpropanoid metabolism, and energy metabolism in MeJA-induced postharvest stress resistance.

## 2 Materials and methods

### 2.1 Plant materials and chemicals

Fresh *G. elata* tubers were harvested in November from Xiaocaoba Town, Zhaotong, Yunnan Province, China (elevation ~1,800–2,300 m). The tubers were cultivated under a local forest-understory imitation-wild cultivation system, with standard practices for *G. elata* in Yunnan: the soil was loose, humus-rich, and slightly acidic (pH 5.5–6.5); the site was shaded by trees (≈70% shade) with ambient temperature ranging 15–25 °C during the growing season and relative humidity of 70–80%. Mushroom sticks (*Armillaria mellea* symbiont, typically made of *Quercus* or *Castanopsis* wood) were buried in the soil to support mycorrhizal symbiosis. No chemical fertilizers or pesticides were applied; weeds were manually removed. Harvesting was conducted on a clear morning, and tubers with similar size and appearance (length 8–12 cm, diameter 3–5 cm) were preliminarily selected in the field immediately after harvest. A total of 300 healthy tubers were initially collected using clean, disinfected hoes and placed in sterile polyethylene bags, then promptly transported to the laboratory. Upon arrival, tubers showing visible insect damage, mechanical injury, or disease symptoms were further excluded, which were used for subsequent experiments. MeJA was purchased from Solarbio (Beijing, China).

### 2.2 Treatment

Uniform tubers were randomly divided into four groups: a control group (deionized water containing 0.05% DMSO) and three MeJA treatment groups (50, 100, and 150 μM MeJA dissolved in deionized water containing 0.05% DMSO). Samples were collected from each treatment at six time points (0, 1, 3, 5, 7, and 9 d), with three biological replicates per time point. Each biological replicate consisted of three tubers pooled together. All tubers were placed in an incubator (24 ± 1 °C, 75–80% relative humidity, in darkness) during the treatment period. At each designated time point, tissues were excised from the equatorial portion of the tubers to a depth of about 4–7 mm. The excised tissues were immediately frozen in liquid nitrogen and stored at −80 °C for subsequent determination of physiological and biochemical indicators.

### 2.3 Determination of respiratory rate, malondialdehyde (MDA) and decay rate

Respiratory activity was monitored using a Dansensor^®^ CheckPoint^®^ 4 gas analyzer (Ametek Mocon, Denmark/USA). At each time point, *G. elata* tubers were enclosed in fresh-keeping bags and held for 40 min before the CO_2_ concentration was determined. Measurements were carried out on storage days 0, 1, 3, 5, 7, and 9. The respiration rate was calculated and expressed as mL mg CO_2_ kg^−1^ h^−1^. For each treatment, three biological replicates were prepared, with eight tubers per replicate.

The determination of MDA content was carried out based on the method of Jin et al. (17). Absorbance readings were recorded at 450, 532, and 600 nm, and MDA levels were presented as mmol kg^−1^.

To evaluate the decay rate, each treatment comprised three replicates, and 100 fresh *G. elata* samples were included in each replicate. The decay rate (%) was calculated using the following formula:


$$\text{Deterioration rate }(\%)\;=\;\frac{\text{Number of decayed samples}}{\text{Total number of samples}}\;\times \;100$$

### 2.4 Determination of O_2_•− production rate, H_2_O_2_ and ROS metabolism-related enzymes activity

Crude enzyme extract for O_2_•− measurement was prepared by homogenizing 1.0 g of tissue with 10 mL of 100 mM phosphate buffer (pH 7.6) containing 0.1% (w/v) PVPP. After centrifugation the supernatant was collected and analyzed following the method described by Lv et al. (22). Data were reported as mmol g^−1^ min^−1^.

H_2_O_2_ content was assayed following Lv et al. (22) with slight modifications. Briefly, 1 g of tissue was extracted with 3 mL of ice-cold acetone and centrifuged at 11,000 × g for 15 min at 4 °C. One milliliter of supernatant was reacted with 100 μL concentrated ammonia and 100 μL 20% titanium tetrachloride. The precipitate obtained after centrifugation was washed with cold acetone, dissolved in 1.5 mL of 1 mM sulfuric acid, and quantified at 410 nm. The results were expressed as μmol g^−1^.

Crude enzyme extracts for SOD, CAT, and POD analyses were prepared by homogenizing the tissue in phosphate buffer (100 mM, pH 7.8) supplemented with DTT and PVPP. Specifically, the extraction medium contained 4.5 mM DTT and 8 g L^−1^ PVPP. After centrifugation, the supernatant was collected for subsequent assays. SOD, CAT, and POD activities were determined using commercial microassay kits (Solarbio Life Science, Beijing, China) following the protocols provided by the manufacturer.

### 2.5 Measurement of AsA and GSH levels and activities of enzymes associated with the AsA–GSH cycle

AsA and GSH contents were measured using commercial kits obtained from Solarbio (Beijing, China) (BC1230 and BC1170, respectively). The results were expressed as mg kg^−1^ for AsA and mmol kg^−1^ for GSH.

APX, GR, monodehydroascorbate reductase (MDHAR), and dehydroascorbate reductase (DHAR) activities were assayed following Tang et al. (23) with slight modifications. Enzyme extracts were prepared in phosphate buffer (pH 7.5). APX, DHAR, and GR were extracted with 0.1 M phosphate buffer containing 1 mM EDTA, whereas MDHAR was extracted using 0.04 M phosphate buffer supplemented with PVPP and β-mercaptoethanol. APX, GR, and MDHAR activities were monitored at 340 nm, and DHAR activity was measured at 290 nm. One unit of enzyme activity was defined as a 0.01 change in absorbance per minute and expressed as U mg^−1^.

### 2.6 Assessment of ATP, ADP, and AMP contents, energy charge, and related enzyme activities in energy metabolism

Adenylates were extracted from frozen tissues using perchloric acid and quantified by HPLC. Briefly, 2.5 g of sample was ground with pre-cooled perchloric acid and centrifuged at 4 °C. The supernatant was neutralized with KOH, adjusted to a fixed volume, and filtered before chromatographic analysis. ATP, ADP, and AMP contents were determined from corresponding standard curves and expressed on a fresh weight basis. The results were expressed as g kg^−1^. The energy charge was calculated using the equation (ATP + 0.5ADP)/ (ATP + ADP + AMP).

Commercial assay kits supplied by Nanjing Jiancheng Bioengineering Institute (Nanjing, China) were used to determine the activities of H^+^-ATPase, Ca^2+^-ATPase, CCO, and SDH, following the protocols provided by the manufacturer. Enzyme activities were expressed as U mg^−1^.

### 2.7 Measurement of phenylpropanoid metabolism-related enzyme activities and metabolite contents

Total phenolics, flavonoids, and lignin contents were determined according to the method of Zhang et al. (13), with slight modifications. For the determination of total phenolics and flavonoids, approximately 1.0 g of frozen *G. elata* tissue was homogenized with pre-cooled 1% (v/v) HCl–methanol at 4 °C and then allowed to stand for 10 min. The homogenate was centrifuged at 11,000 × g for 10 min at 4 °C, and the supernatant was collected. The absorbance was measured at 280 and 325 nm for the analysis of total phenolics and flavonoids, respectively. Total phenolics were expressed as mmol kg^−1^, while flavonoids were expressed as g kg^−1^.

For lignin determination, approximately 1.0 g of frozen tissue was homogenized with pre-cooled 95% ethanol at 4 °C and then centrifuged. The resulting pellet was sequentially washed with ethanol and ethanol solution at a ratio of 1:2 (v/v), followed by drying. The dried residue was reacted with acetic acid containing 25% (v/v) acetyl bromide. After the reaction, NaOH, 1.0 mL acetyl bromide, and 0.05 mL hydroxylamine hydrochloride were added sequentially. The mixture was centrifuged at 11,000 × g for 10 min, and the absorbance of the final supernatant was measured at 280 nm. Lignin content was expressed as g kg^−1^.

The extraction of PAL, C4H, 4CL, and CAD was performed from 1.0 g of frozen tissue using individual pre-cooled buffers, and the crude enzyme extracts were collected after centrifugation at 4 °C. Activities of these enzymes were assayed following Deng et al. (24). PAL activity was determined at 290 nm with L-phenylalanine as substrate; C4H activity was monitored at 340 nm; 4CL activity was measured at 333 nm in a system containing crude enzyme extract, MgCl_2_, coenzyme A, p-coumaric acid, and ATP; and CAD activity was evaluated at 340 nm by measuring NADPH oxidation in the presence of cinnamaldehyde. Enzyme activities were expressed as U mg^−1^, where one unit was defined as a 0.01 absorbance change per minute.

### 2.8 RNA extraction and quantitative real-time PCR analysis

Total RNA was isolated using the NucleoSpin RNA Plant kit (Macherey-Nagel, Germany), and first-strand cDNA was synthesized from 1 μg of total RNA using the QuantiNova Reverse Transcription Kit (Qiagen, Germany). RT-qPCR was performed on a CFX96 Touch Real-Time PCR Detection System (Bio-Rad, USA) in 20 μL reaction mixtures containing 1 μL of cDNA template, 1 μL each of 10 μM forward and reverse primers, and 10 μL of SYBR™ Green qPCR Master Mix. *GeActin* was used as the internal reference gene. Relative expression levels were calculated using the 2^−Δ*Δt*^ method. Primer sequences are listed in Table S1.

### 2.9 Data analysis

Differences among groups were evaluated by two-way analysis of variance (ANOVA), and mean separation was subsequently performed using Duncan's multiple range test at *p* < 0.05. Uppercase letters represent significant differences among storage times within the same treatment, whereas lowercase letters indicate significant differences between treatments at the same storage time (*p* < 0.05).

## 3 Results

### 3.1 Influence of MeJA on visual appearance, decay, respiratory rate and MDA in *G. elata*

**Figure 1:**
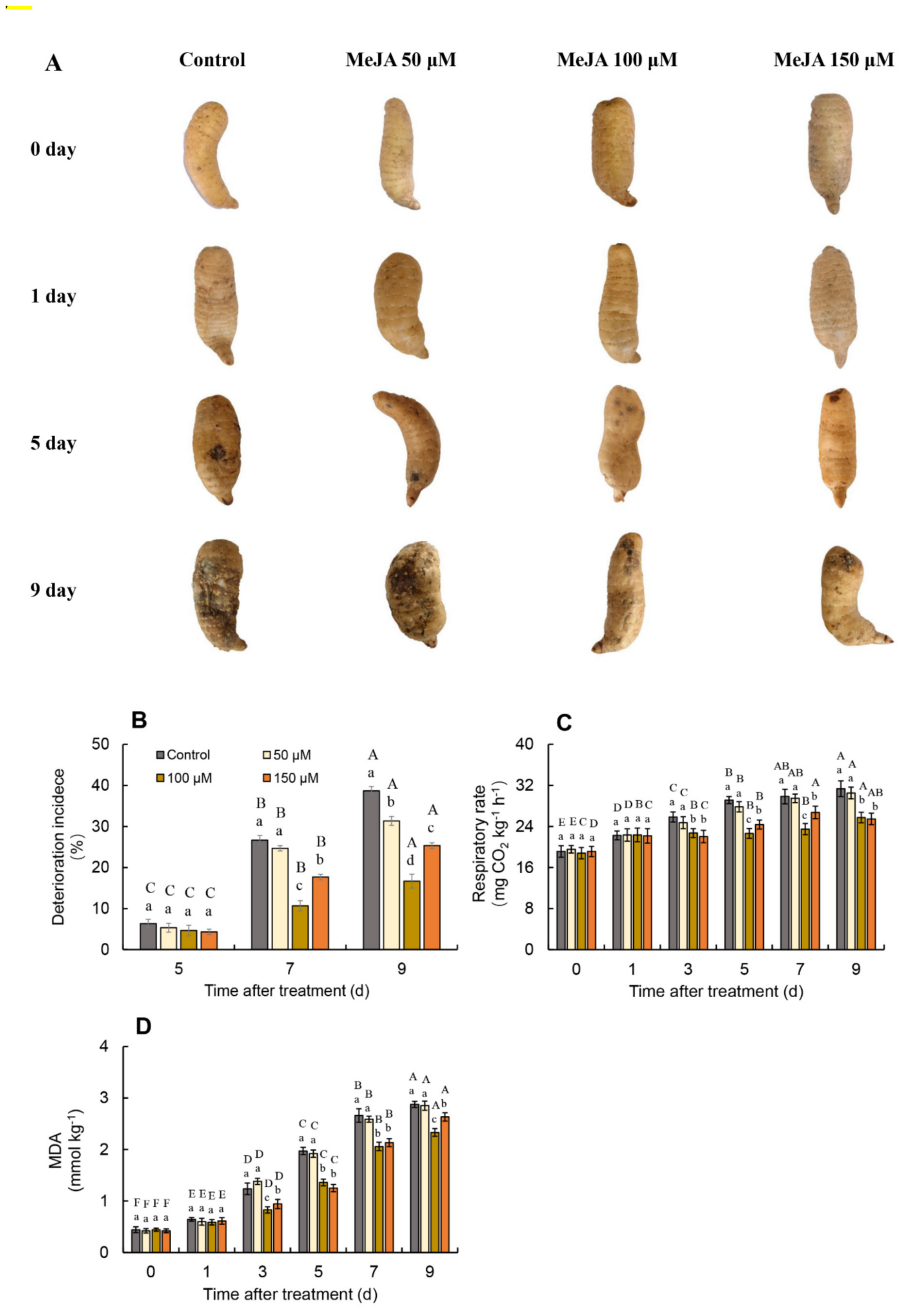
Visual appearance of fresh *G. elata* tubers after treatment with different concentrations of methyl jasmonate (MeJA) during storage **(A)**. Effect of MeJA treatment on the decay incidence **(B)**, respiratory rate **(C)** and malondialdehyde (MDA) **(D)** in *Gastrodia elata*. Capital letters represent significant differences among storage times within the same treatment, whereas lowercase letters indicate significant differences between treatments at the same storage time (*p* < 0.05). Vertical bars represent the standard errors of the means (±SE).

Phenotypic observations showed that fresh *G. elata* tubers maintained a relatively intact appearance at 0 and 1 d of storage. As storage progressed, the control tubers began to show visible decay symptoms at 5 d and developed severe rot by 9 d. Compared with the other MeJA concentrations, treatment with 100 μM MeJA maintained better visual quality at 9 d, as indicated by less surface decay (Figure 1A).

No visible decay was detected in either the MeJA-treated group or the control in the initial storage period (0–3 d). However, by days 7–9, the decay incidence of *G. elata* tubers treated with 100 μM MeJA was markedly lower relative to the control and lower than that observed under the other MeJA concentrations (Figure 1B). Similarly, MeJA treatment effectively suppressed the respiration rate and MDA accumulation in *G. elata*. As shown in Figures 1C, D, the 100 μM MeJA treatment consistently produced better results than the other concentrations. Accordingly, 100 μM MeJA was chosen for the following experiments.

### 3.2 Effect of MeJA on ROS metabolism

**Figure 2:**
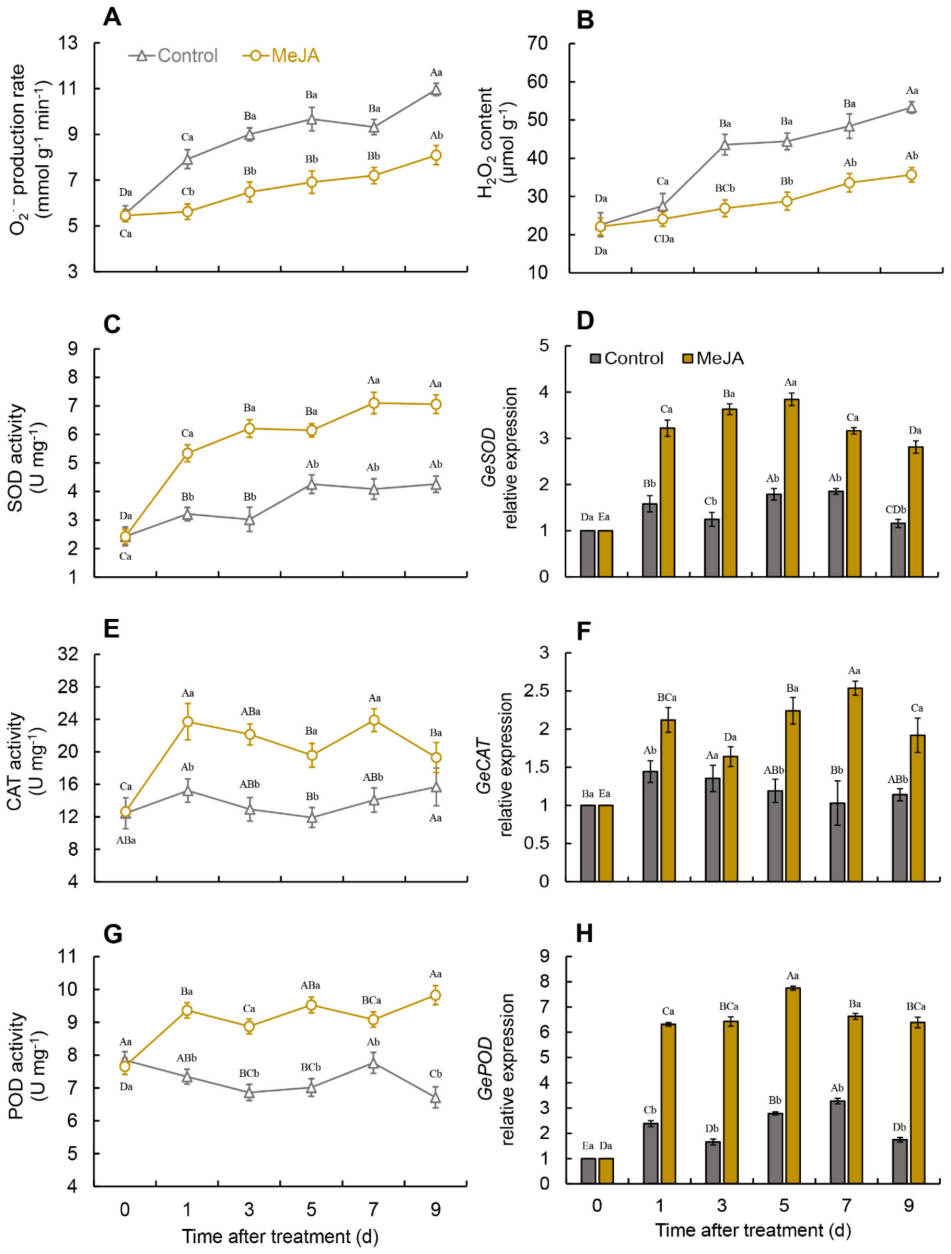
Changes in anion generation (O_2_•−), **(A)** and hydrogen peroxide (H_2_O_2_), **(B)**, and the activities and expressions of superoxide dismutase (SOD) **(C, D)**, catalase (CAT) **(E, F)** and peroxidase (POD) **(G, H)** in *Gastrodia elata* after methyl jasmonate (MeJA) treatment during storage at room temperature. Capital letters represent significant differences among storage times within the same treatment, whereas lowercase letters indicate significant differences between treatments at the same storage time (*p* < 0.05). Vertical bars represent the standard errors of the means (±SE).

As shown in Figure 2A, throughout storage, the control group consistently exhibited a higher O_2_•− production rate than the MeJA-treated group. The MeJA-treated samples exhibited a similar H_2_O_2_ level to the control during the initial stage of storage (0–1 d), but it was reduced relative to the control from 3 to 9 d (Figure 2B). During storage, SOD activity in the MeJA-treated group rose rapidly during 0–3 d, then fluctuated thereafter, but displayed an overall increasing trend and remained consistently above the control level throughout trial stage. The expression level of *GeSOD* in the treated group increased sharply during 0–5 d, reached its maximum level, and then declined (Figure 2C). In contrast, *GeSOD* expression in the control showed fluctuations over the storage period and remained below the level observed in the treated group (Figure 2D). CAT activity in the MeJA-treated group exhibited two distinct peaks at 1 and 7 d and remained markedly above the control level from 1 to 7 d (Figure 2E). Relative to the control, MeJA treatment promoted *GeCAT* expression at 1 d and from 5 to 9 d (Figure 2F). A significant rise in POD activity was noted in the MeJA-treated group during 0–1 d, after which the activity fluctuated during 3–9 d, nevertheless, it remained greater than the level observed in the control throughout the experiment (Figure 2G). Similar to POD activity, MeJA also effectively enhanced *GePOD* expression during storage (Figure 2H).

### 3.3 Effect of MeJA on AsA–GSH cycle

**Figure 3:**
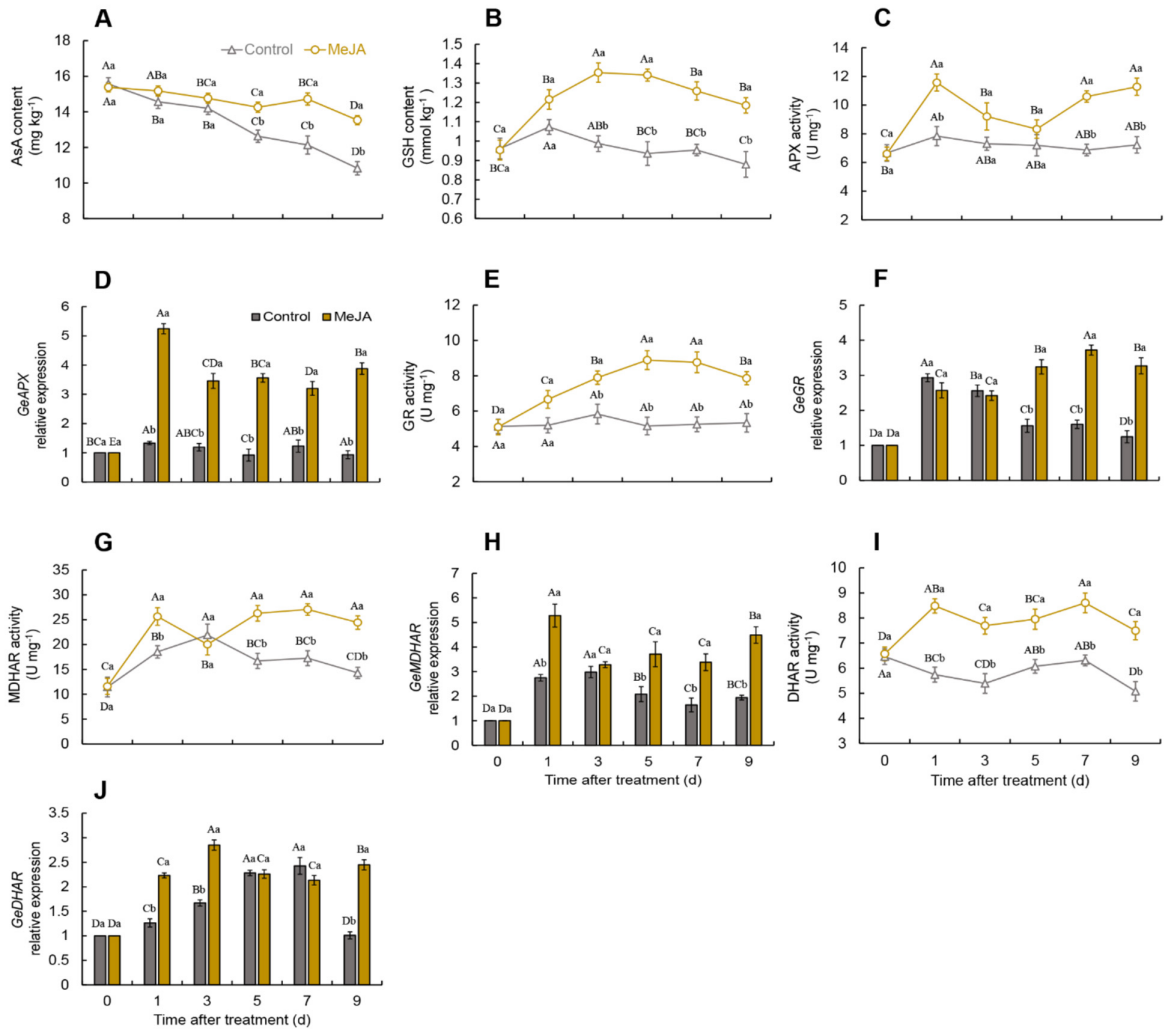
Changes in ascorbic acid (AsA) **(A)** and glutathione (GSH) **(B)** contents. and the activities and expressions of ascorbate peroxidase (APX) **(C, D)**, glutathione reductase (GR) **(E, F)**, monodehydroascorbate reductase (MDHAR) **(G, H)**, and dehydroascorbate reductase (DHAR) **(I, J)** in *Gastrodia elata* after methyl jasmonate (MeJA) treatment during storage at room temperature. Capital letters represent significant differences among storage times within the same treatment, whereas lowercase letters indicate significant differences between treatments at the same storage time (*p* < 0.05). Vertical bars represent the standard errors of the means (±SE).

AsA content did not differ significantly between the two treatments in the early storage period (0–3 d). However, from 5 to 9 d, the AsA content was elevated in the treated group (Figure 3A). As shown in Figure 3B, GSH content rose rapidly during 0–3 d following MeJA treatment and then declined, but remained elevated relative to the control from 3 to 9 d. According to Figure 3C, APX activity changed only slightly in the control group throughout storage, whereas the MeJA-treated group displayed fluctuations and was greater than the control at 1 d and during 7–9 d. In addition, The MeJA-treated group exhibited remained elevated *GeAPX* expression than the control throughout storage (Figure 3D). GR activity in the MeJA-treated group rose continuously during 0–5 d, reached its highest level, and then declined, but still stayed above the control during 3–9 d. By contrast, GR activity in the control showed little variation over the entire storage period (Figure 3E). No marked difference in *GeGR* expression was observed between the treated and control groups during 0–3 d; however, during the middle and late storage stages (5–9 d), the treated group exhibited higher *GeGR* expression than the control (Figure 3F). MeJA treatment caused a pronounced rise in the activity and transcription levels of MDHAR in *G. elata* tubers at 1 d and 5–9 d (Figures 3G, H). From 1 to 9 d, DHAR activity showed a comparable pattern in both the control and MeJA-treated groups, but remained consistently greater in the treated group throughout storage (Figure 3I). Moreover, MeJA treatment enhanced *GeDHAR* expression at 1–3 d and 9 d (Figure 3J).

### 3.4 Effect of MeJA on energy metabolism

**Figure 4:**
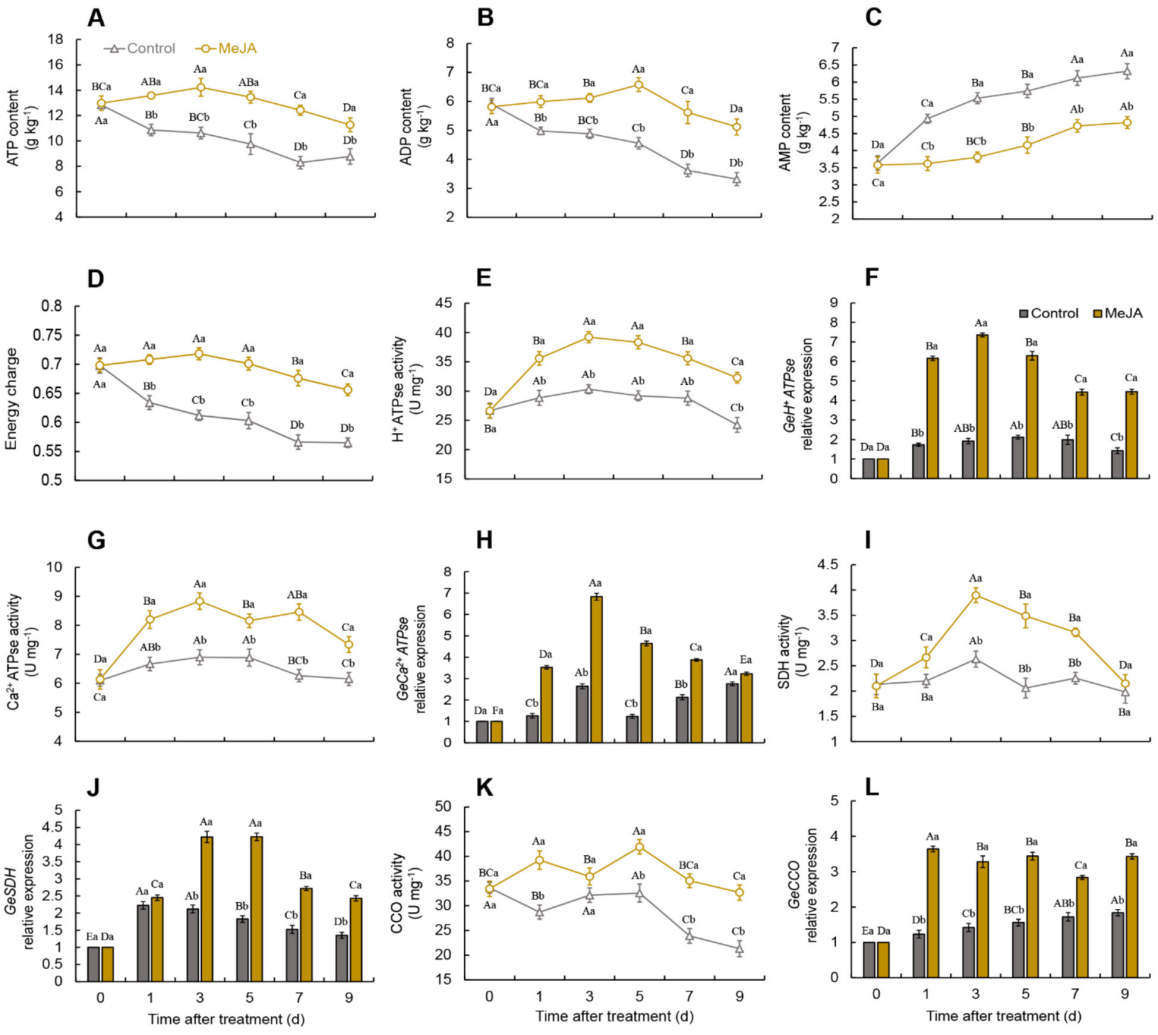
Changes in the content of ATP **(A)**, ADP **(B)**, AMP **(C)** and energy charge **(D)**, and the activities and expressions of H^+^-ATPase **(E, F)**, Ca^2+^-ATPse **(G, H)**, succinate dehydrogenase (SDH) **(I, J)** and cytochrome c oxidase (CCO) **(K, L)** in *Gastrodia elata* after methyl jasmonate (MeJA) treatment during storage at room temperature. Capital letters represent significant differences among storage times within the same treatment, whereas lowercase letters indicate significant differences between treatments at the same storage time (*p* < 0.05). Vertical bars represent the standard errors of the means (±SE).

According to Figures 4A–C, after MeJA treatment, the ATP and ADP contents in *G. elata* tubers were maintained at levels above those of the control, whereas AMP content remained below the control level. Meanwhile, the treated group also exhibited a consistently greater energy charge than the control group (Figure 4D). The activity and expression of H^+^-ATPase in the MeJA-treated group followed similar trends, both rising rapidly during 0–3 d, reaching a maximum, and then declining. Even so, both the activity and transcript level of *GeH*^+^*-ATPase* remained above those in the control throughout storage (Figures 4E, F). In the control group, Ca^2+^-ATPase activity rose slightly during 0–5 d and then declined, whereas in the MeJA-treated group, it displayed two peaks at 3 and 7 d and stayed above the control level throughout the experiment (Figure 4G). The expression of *GeCa*^2+^*-ATPase* in the MeJA-treated group reached its maximum at 3 d and exceeded that in the control during 1–5 d (Figure 4H). SDH activity in the MeJA-treated group rose sharply during 0–3 d, reached its highest value, and then declined, whereas the control group showed a fluctuating pattern over the experimental period. Moreover, the treated group exhibited greater SDH activity during 3–7 d (Figure 4I). The expression of *GeSDH* in the MeJA-treated group also remained above that in the control from 3 to 9 d (Figure 4J). CCO activity in the control group was relatively constant during 0–5 d, followed by a decline. By contrast, CCO activity in the MeJA-treated group varied throughout the experimental period and surpassed the control at 1 d and 5–9 d (Figure 4K). Following MeJA treatment, *GeCCO* expression was maintained at a higher level than in the control (Figure 4L).

### 3.5 Effect of MeJA on phenylpropanoid pathway

**Figure 5:**
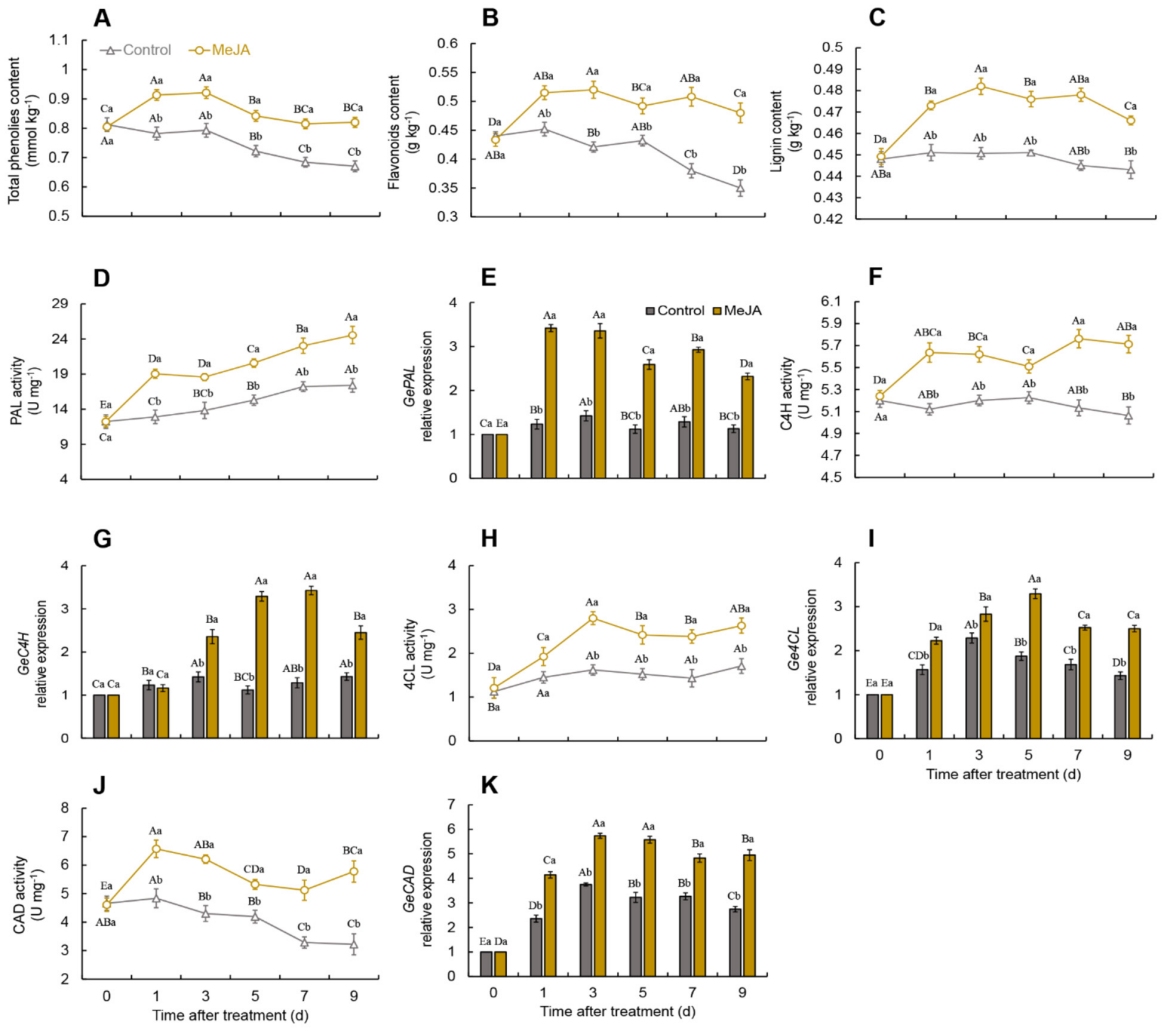
Changes in the content of total phenols **(A)**, flavonoids **(B)** and lignin **(C)**, and the activities and expressions of phenylalanine ammonia-lyase (PAL) **(D, E)**, cinnamate 4-hydroxylase (C4H) **(F, G)**, 4-coumarate ligase (4CL) **(H, I)** and cinnamyl alcohol dehydrogenase (CAD) **(J, K)** in *Gastrodia elata* after methyl jasmonate (MeJA) treatment during storage at room temperature. Capital letters represent significant differences among storage times within the same treatment, whereas lowercase letters indicate significant differences between treatments at the same storage time (*p* < 0.05). Vertical bars represent the standard errors of the means (±SE).

According to Figures 5–C, MeJA application resulted in elevated levels of total phenolics, flavonoids, and lignin in *G. elata* tubers compared with the control. An overall rise in PAL activity was noted in both the control and MeJA-treated groups, whereas PAL activity in the treated group remained above the control level throughout the whole experiment (Figure 5D). In addition, *GePAL* expression in the control showed little variation during 0–9 d, whereas in the treated group it rose sharply during 0–1 d and then declined, while still remaining above the control level (Figure 5E). C4H activity in the control group showed an overall downward trend, whereas the treated group exhibited an increasing trend and maintained higher activity throughout storage relative to the control (Figure 5F). Following MeJA treatment, *GeC4H* expression was elevated during 3–9 d (Figure 5G). The changes in 4CL activity followed a similar pattern in both groups, increasing during 0–3 d and fluctuating thereafter. However, from 3 to 9 d of storage, 4CL activity in the treated group remained above that in the control (Figure 5H). Moreover, MeJA treatment promoted *Ge4CL* expression throughout the experimental period (Figure 5I). Compared with the control, MeJA treatment also effectively increased both CAD activity and its expression level in *G. elata* tubers (Figures 5J, K).

### 3.6 Transcription levels of MYB, NAC and WRKY TFs genes

**Figure 6:**
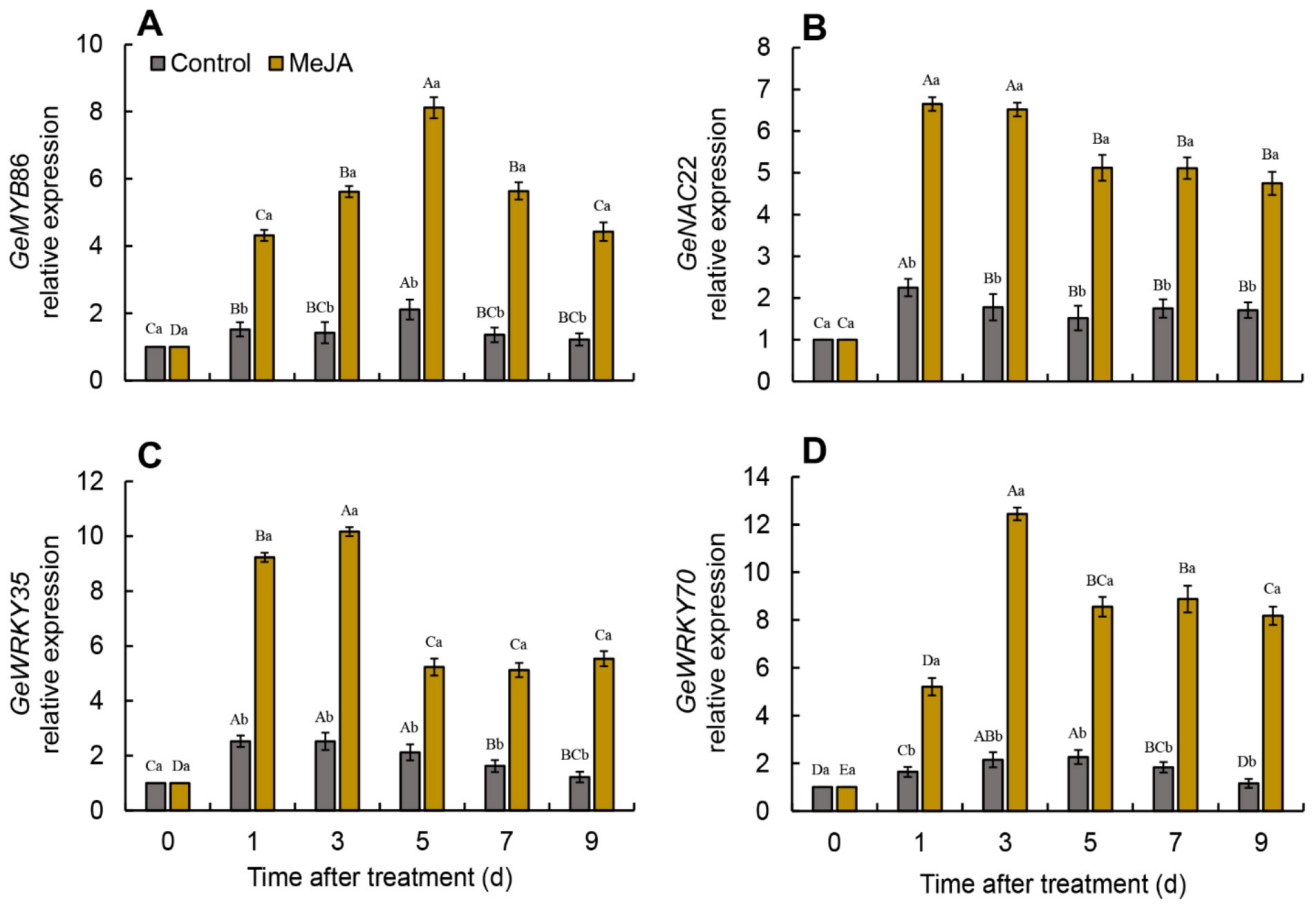
Changes in expressions of *GeMYB86* **(A)**, *GeNAC22* **(B)**, *GeWRKY35* **(C)** and *GeWRKY70* **(D)** in *Gastrodia elata* after methyl jasmonate (MeJA) treatment during storage at room temperature. Capital letters represent significant differences among storage times within the same treatment, whereas lowercase letters indicate significant differences between treatments at the same storage time (*p* < 0.05). Vertical bars represent the standard errors of the means (±SE).

As shown in the Figures 6A–D, the transcript levels of *GeMYB86, GeNAC22*, and *GeWRKY35/70* were maintained at higher levels in the MeJA-treated group than in the control over the course of storage.

## 4 Discussion

Enhanced respiratory metabolism and aggravated membrane lipid peroxidation are important factors leading to postharvest senescence, decay, and quality deterioration in fruits and vegetables (25, 26). Excessive respiration accelerates substrate consumption and tissue senescence, whereas MDA, which is generated during membrane lipid peroxidation, is widely regarded as a marker of membrane damage, which can further promote disease development and tissue decay. Therefore, suppressing abnormal respiration, reducing membrane lipid peroxidation, and alleviating decay are important approaches for maintaining postharvest quality. In this study, MeJA treatment markedly decreased the respiration rate, decay incidence, and MDA accumulation in fresh *G. elata*, suggesting that MeJA contributes to the retardation of respiratory metabolism, the reduction of lipid peroxidative damage, and the preservation of membrane integrity. Comparable findings have likewise been documented in other postharvest studies. For example, MeJA treatment lowered the respiration rate of peach fruit and effectively suppressed MDA accumulation, thereby maintaining better fruit quality (27). In apples, MeJA dipping treatment significantly suppressed respiration intensity and helped preserve storage quality (20). In addition, in sweet cherry, MeJA treatment enhanced resistance to *Alternaria alternata*-induced decay (28), and significantly inhibited lesion expansion in mango while reducing MDA content (29).

Abiotic stress can induce a rapid buildup of ROS in plant tissues. Low concentrations of ROS are important for signaling and defense activation in plants, whereas excessive ROS accumulation is detrimental and results in oxidative injury (30, 31). Plants possess an integrated antioxidant defense system composed of key antioxidant enzymes, including SOD and CAT, combined with the AsA–GSH cycle, which functions to eliminate excess ROS and maintain ROS homeostasis (32). POD also participates in a variety of cellular processes, including H_2_O_2_ scavenging, phenol oxidation, and lignification (6, 33). In this study, MeJA treatment lowered the O_2_•− production rate and H_2_O_2_ content in *G. elata* tubers relative to the control. Moreover, MeJA promoted SOD, CAT, and POD activity and elevated the transcript abundance of the corresponding genes. MeJA treatment in loquat fruit was found to reduce chilling injury while enhancing antioxidant enzyme activities (34). In peach fruit, MeJA significantly reduced ROS accumulation and maintained better quality by enhancing the AsA–GSH cycle and antioxidant capacity (27). A similar phenomenon has been observed in mango fruit, where MeJA application suppressed ROS production and promoted antioxidant enzyme activities, thus alleviating chilling injury (35). Collectively, the beneficial role of MeJA in *G. elata* tubers appears to be associated with improved antioxidant enzyme activity, which may help preserve cellular redox balance.

In harvested fruits and vegetables, the AsA–GSH cycle serves as a key antioxidant pathway for scavenging ROS and maintaining cellular redox homeostasis (36). APX participates in this cycle by reducing H_2_O_2_ with AsA serving as the electron donor, while GR sustains GSH regeneration to ensure the proper functioning of the cycle. In this pathway, AsA and GSH function as the principal non-enzymatic antioxidants. Upon oxidation, AsA is converted into MDHA and DHA; MDHA can be regenerated back to AsA by MDHAR, while DHAR reduces DHA to AsA in a GSH-dependent manner (37, 38). Consequently, APX, GR, MDHAR, and DHAR, together with AsA and GSH, cooperate in the efficient removal of excess ROS, thereby alleviating oxidative injury. It was found that MeJA treatment led to the accumulation of AsA and GSH and promoted APX, GR, DHAR, and MDHAR enzyme activities and transcript abundance in *G. elata*. As reported by Zhu et al. (27), MeJA treatment increased the activities of APX, GR, MDHAR, and DHAR and preserved higher AsA and GSH contents in peach fruit. Comparable observations have also been reported in studies involving MeJA-treated tomato (39) and loquat fruit (40). The present findings suggest that MeJA treatment may stimulate the AsA–GSH cycle by promoting the activities and transcription levels of APX, GR, MDHAR, and DHAR, thereby increasing AsA and GSH accumulation, promoting the detoxification of excess ROS in *G. elata*, and maintaining ROS homeostasis.

In plants, the phenylpropanoid pathway constitutes a major secondary metabolic route and is primarily responsible for the biosynthesis of phenolic compounds. The principal rate-limiting enzymes in this pathway include PAL, 4CL, and C4H (41). PAL functions as the first rate-limiting enzyme in the phenylpropanoid pathway and mediates the conversion of L-phenylalanine to cinnamic acid, after which C4H and 4CL further catalyze the formation of corresponding thioesters from different hydroxycinnamic acids, which then enter the biosynthetic pathways of phenolics, flavonoids, anthocyanins, and other compounds (42). Phenolic compounds are not only precursors for the synthesis of disease-resistant substances such as lignin and possess certain antimicrobial properties, but also facilitate lignin deposition in the cell wall (43). Our findings indicated that, during storage, MeJA application in *G. elata* resulted in elevated PAL, C4H, and 4CL activities together with increased transcript abundance of these genes, and was accompanied by greater accumulation of total phenolics, flavonoids, and lignin. In tomato fruit, exogenous MeJA treatment promoted the transcript levels of phenylpropanoid metabolism-related genes, including PAL, C4H, and 4CL, enhanced the activities of the corresponding enzymes, and increased the accumulation of total phenolics and flavonoids, suggesting that MeJA stimulates phenolic biosynthesis through activation of the phenylpropanoid pathway (44). Accumulating evidence suggests that MeJA treatment can enhance wound-induced defense responses in fresh-cut pitaya, increased the activities and transcription of phenylpropanoid metabolism-related enzymes, and favor the accumulation of phenolics (45). Similarly, in postharvest kiwifruit, MeJA treatment maintained phenolic content by upregulating the expression of PAL and C4H, indicating that MeJA-induced quality maintenance and disease resistance are closely associated with activation of the phenylpropanoid pathway (31). In studies on wound healing in postharvest kiwifruit, MeJA treatment was also demonstrated to elevate the activities and transcript abundance of PAL, C4H, 4CL, and PPO, while enhancing the buildup of suberin polyphenolics and lignin-related substances, which contributed to accelerated wound healing (46). Based on these observations, it is reasonable to propose that MeJA activates the phenylpropanoid pathway, thus accelerating the buildup of total phenolics, flavonoids, and lignin and consequently improving the antioxidant defense and storage performance of fresh *G. elata*.

Importantly, the maintenance of phenylpropanoid metabolism and cellular energy status in this study not only contributes to postharvest quality preservation of *G. elata*, but also has important implications for its functional food-related value. Phenolic compounds and flavonoids are well recognized for their strong antioxidant, anti-inflammatory, and bioactivity-related properties, which are closely associated with human nutraceutical benefits (47). Therefore, the MeJA-induced accumulation of these bioactive compounds may help preserve the functional quality and nutritional potential of *G. elata* during storage. In parallel, the maintenance of energy metabolism contributes to cellular integrity and may delay the degradation of these bioactive metabolites (48). Collectively, these findings suggest that postharvest treatment not only affects physiological quality but also plays a key role in preserving the nutraceutical value and health-related quality of fresh *G. elata*.

ATP is recognized as the major energy source required for crucial biological functions. Numerous studies have shown that energy generated through cellular metabolism is closely involved in the regulation of senescence, quality, and abiotic stress resistance in harvested fruits (49). In addition, membrane integrity is essential for normal cellular function. However, ATP deficiency can make plants more susceptible to membrane damage under stress, which in turn leads to further ROS-induced membrane injury (50). By contrast, a higher energy status helps maintain membrane integrity and improves postharvest quality. The current study, MeJA treatment elevated the energy status of fresh postharvest *G. elata*. Jin et al. (51) reported that MeJA treatment significantly increased ATP and ADP contents while decreasing AMP content in peach fruit, thereby maintaining a higher energy charge. Similar results have also been observed in studies on *ginger rhizomes* (8) and ‘Nanguo' pear fruit (19).

The preservation of cellular energy homeostasis largely depends on mitochondrial enzyme activity. H^+^-ATPase and Ca^2+^-ATPase contribute to energy-dependent ion transport, while SDH and CCO are central to oxidative phosphorylation and ATP biosynthesis in plants (52). In this study, MeJA treatment significantly improved H^+^-ATPase, Ca^2+^-ATPase, SDH, and CCO activities and the expression of their related genes in *G. elata* tubers. Huang et al. (35) similarly found in mango fruit that MeJA treatment not only increased intracellular ATP levels, but also enhanced the activities of IDH, α-KGDH, SDH, CCO, H^+^-ATPase, and Ca^2+^-ATPase, thereby maintaining energy homeostasis, alleviating chilling injury, and preserving postharvest quality. The above results indicate that MeJA treatment is beneficial for maintaining the postharvest energy status of fresh *G. elata*. An adequate energy supply may help sustain normal metabolic processes, postpone storage-related senescence, and consequently preserve postharvest quality more effectively.

In addition, a growing body of evidence indicates that MYB, NAC, and WRKY belong to transcription factor families with important regulatory functions in regulating phenylpropanoid metabolism. MYB is able to directly regulate phenylpropanoid pathway-related genes (53). NAC is often involved in the upstream regulation of lignin biosynthesis, and WRKY promotes the activation of phenylpropanoid metabolism and related secondary metabolic pathways during stress responses and defense reactions (54, 55). This study preliminarily identified four transcription factors belonging to the MYB, NAC, and WRKY families through RNA-seq transcriptomic analysis. The results showed that, following MeJA treatment, these transcription factors, including *GeMYB86, GeNAC22*, and *GeWRKY35/70*, were all upregulated. Previous studies have also demonstrated that MYB, NAC, and WRKY transcription factors play key regulatory roles in fruit phenylpropanoid metabolism and defense responses. For example, in peach fruit, *PpWRKY70* can directly activate the transcription of *PpPAL* and *Pp4CL*, thereby enhancing phenylpropanoid metabolism and improving resistance to *Rhizopus stolonifer* (56). In loquat fruit, *EjNAC1* has been shown to transcriptionally activate lignin biosynthesis-related genes and promote postharvest fruit lignification (57). In addition, in navel orange fruit, transcriptomic analysis during H_2_S-induced disease resistance suggested that MYB and WRKY transcription factors may participate in the regulation of phenylpropanoid metabolism and are closely associated with enhanced disease resistance (58). Overall, in *G. elata* tubers, MeJA treatment induced higher expression of MYB, NAC, and WRKY transcription factors, suggesting that these factors may enhance the accumulation of total phenolics, flavonoids, and lignin by regulating genes associated with the phenylpropanoid pathway, thereby helping preserve postharvest quality. Although this study preliminarily revealed the potential roles of MYB, NAC, and WRKY in MeJA-induced phenylpropanoid metabolism in *G. elata*, the underlying regulatory mechanisms still require follow-up research.

Compared with other commonly used postharvest preservation agents or strategies, MeJA exhibits a distinctive signal-elicitation regulatory feature in fresh *G. elata* (59, 60). Melatonin is generally associated with antioxidant protection and ROS homeostasis (21), chitosan-based coatings mainly reduce water loss and pathogen invasion through physical barrier formation and antimicrobial effects (61), and low-temperature storage delays quality deterioration primarily by suppressing respiration and metabolic activity (62). In contrast, MeJA is a jasmonate-derived defense signaling molecule that may act as a pre-storage elicitor to mobilize endogenous resistance before severe deterioration occurs (60). In the present study, MeJA treatment concurrently reduced decay incidence, respiration rate, and membrane lipid peroxidation, while enhancing antioxidant defense, the AsA–GSH cycle, phenylpropanoid metabolism, and energy metabolism. Therefore, the distinctive regulatory advantage of MeJA may lie in its inducible and multi-pathway regulatory mode, which integrates redox homeostasis, defense-related secondary metabolism, and cellular energy supply to maintain the commercial and functional quality of fresh *G. elata*.

Among these preservation agents, melatonin is particularly relevant to the present study. Dong et al. (21) reported that melatonin maintained the postharvest quality of fresh *G. elata* tubers by regulating antioxidant ability, phenylpropanoid metabolism, and energy metabolism during storage. Consistently, MeJA treatment in this study also delayed postharvest deterioration by regulating ROS metabolism, phenylpropanoid metabolism, and energy metabolism. These results suggest that melatonin and MeJA may share several downstream physiological processes related to postharvest quality maintenance. However, their regulatory characteristics are different. Melatonin-mediated quality preservation is more closely associated with antioxidant protection, ROS homeostasis, and stress alleviation, whereas MeJA functions more prominently as a jasmonate-derived defense signal and elicitor of endogenous resistance (59, 60). In particular, the expression changes of selected MYB, NAC, and WRKY transcription factors observed in this study provide preliminary clues that MeJA may be involved in broader transcriptional regulation associated with postharvest stress adaptation. Thus, compared with the previously reported melatonin-mediated antioxidant protection mechanism, the present study provides a jasmonate-related defense regulatory perspective for understanding postharvest quality preservation in fresh *G. elata*.

From an industrial perspective, these findings suggest that MeJA can serve as a pre-storage elicitor to enhance intrinsic stress resistance and physiological stability of fresh *G. elata* tubers, rather than functioning merely as a postharvest preservative. This distinction is important: unlike conventional chemical preservatives that act directly, MeJA triggers endogenous defense mechanisms, potentially offering longer-lasting and more sustainable protection. For practical application, MeJA treatment could be integrated with low-temperature storage, humidity control, appropriate packaging, and cold-chain logistics to maximize effectiveness. However, key parameters such as treatment concentration, immersion duration, packaging compatibility, cost-effectiveness, and stability under large-scale distribution conditions still require systematic optimization. Therefore, this study provides a physiological basis and technical reference for developing MeJA-based green preservation strategies tailored to the fresh *G. elata* industry.

## 5 Conclusions

**Figure 7:**
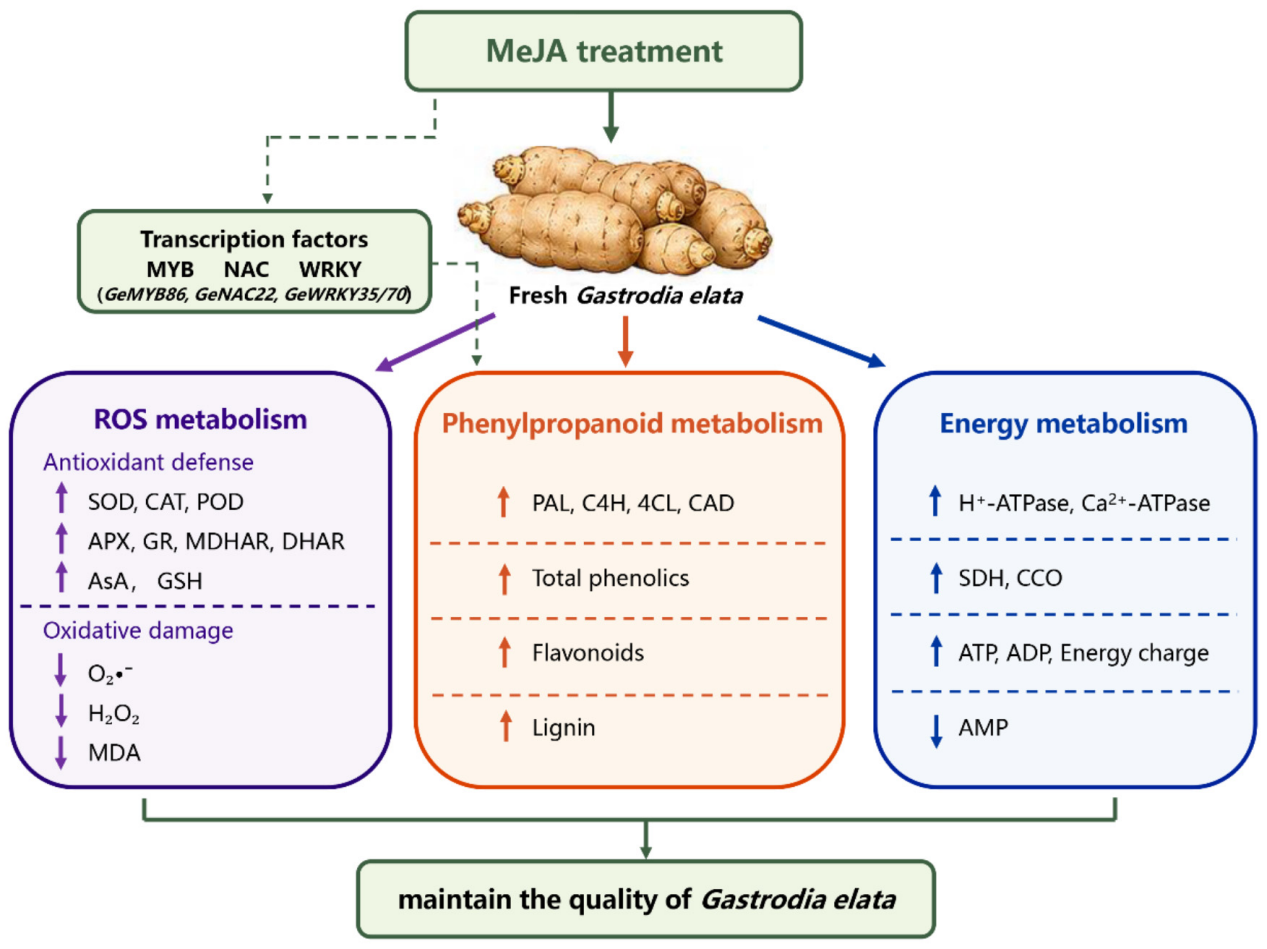
A proposed mechanism illustrating the role of methyl jasmonate (MeJA) in maintaining the postharvest quality of *Gastrodia elata* through the regulation of ROS, phenylpropanoid, and energy metabolism.

To conclude, the current findings indicate that exogenous 100 μM MeJA exhibited the best preservation to enhance the activities of ROS-scavenging enzymes in fresh *G. elata*, thereby reducing ROS-induced cellular damage. Meanwhile, MeJA promoted phenylpropanoid metabolism by regulating the activities of related enzymes and stimulating the accumulation of phenolic compounds, which contributed to the maintenance of antioxidant capacity. MeJA also improved the energy status of fresh *G. elata*, thus facilitating the preservation of its postharvest quality. In addition, MeJA markedly upregulated the expression of MYB, NAC, and WRKY transcription factors, suggesting that these regulators may participate in the activation of phenylpropanoid metabolism and thus contribute to postharvest quality maintenance in *G. elata* (Figure 7). However, the present work has limitations. Although the optimal concentration (100 μM) was identified under standard room temperature conditions, the regulatory effects under different storage environments (e.g., low temperature, controlled atmosphere) remain to be elucidated. Future studies should focus on: (1) validating the efficacy of 100 μM MeJA under various storage conditions to assess practical applicability; (2) exploring the synergistic effects of MeJA with other physical or chemical preservation methods; and (3) integrating the validated transcription factor–target gene pairs and protein interaction data into a tentative model wherein MeJA upregulates *GeMYB, GeNAC*, and *GeWRKY* transcription factors, thereby activating phenylpropanoid metabolism-related genes (e.g., *GePAL, Ge4CL, GeC4H, GeCAD*), leading to enhanced flavonoid and lignin accumulation and consequently improved disease resistance and quality retention. Such investigations will facilitate the commercial postharvest management of *G. elata* and other fresh produce.

## Data Availability

The datasets presented in this study can be found in online repositories. The names of the repository/repositories and accession number(s) can be found in the article/Supplementary material.
