# Supplementary material for: Methyl jasmonate maintains postharvest quality of Gastrodia elata by coordinately regulating ROS homeostasis, phenylpropanoid biosynthesis, and energy metabolism
Source: Front Nutr. 2026 Jul 29;13:1873026. doi: 10.3389/fnut.2026.1873026 (PMC13461387; doi:10.3389/fnut.2026.1873026)
Supplement: Supplementary file 1 [file Table_1.docx]

Supplementary Material

# 1 Supplementary Figures and Tables

**Table S1** Primer sequences used for real-time PCR.

| Gene | Primer sequence | GenBank ID |
| --- | --- | --- |
| *GeSOD* | Forward: GAATGGCCCGACCACAGTTA | GWHGAOSR003300 |
|  | Reverse: CCACGTTTCCCAGATCACCA |  |
| *GeCAT* | Forward: CATGGTGCATGCCCTCAAAC | GWHGAOSR001975 |
|  | Reverse: CCGTCCATGTGCCTGTAGTT |  |
| *GePOD* | Forward: AGACCAAGCGCTCTTCAACA | GWHGAOSR014834 |
|  | Reverse: AGTTCAACCGTATCTGGCCG |  |
| *GeAPX* | Forward: GAGGGCCCATGGACATCAAA | GWHGAOSR005801 |
|  | Reverse: TGCTTCAGCGTAGTCAGCAA |  |
| *GeGR* | Forward: AGACTGCAGCTGAGGCTTTT | GWHGAOSR010328 |
|  | Reverse: TGAGGCATTAGGGCAAGCAA |  |
| *GeMDHAR* | Forward: GAGGCTTGCTCCAGAATGGT | GWHGAOSR004984 |
|  | Reverse: TGTAACCGTGGAACCTGTGG |  |
| *GeDHAR* | Forward: CCCTTGCCCCCAAGCTATAC | GWHGAOSR000302 |
|  | Reverse: GAGCGAAGTCCATCCGACAA |  |
| *GeH^+^-ATPase* | Forward: TTCCTGCAGATGCACGTCTT | GWHGAOSR002053 |
|  | Reverse: TCCACAAGATGGGCTGCTTT |  |
| *GeCa^2+^-ATPase* | Forward: ACCTCAAATGCCCTGAGGTG | GWHGAOSR014108 |
|  | Reverse: AGCCCGATAAAGCCGACAAA |  |
| *GeSDH* | Forward: GAGGTGCTCAAGGAAAGGCT | GWHGAOSR001391 |
| *GeCCO*  *GePAL* | Reverse: TGCAGCCATTAAGCCAGGAA  Forward: CTGGGCATATCGACGCTTCT  Reverse: TCGAAGATTGGGTGGCAGAC  Forward: CCAGACGAAAGGCACTCCAT  Reverse: TCGTCTTCTTTTGCCGGAGT | GWHGAOSR004087  GWHGAOSR000956 |
| *GeC4H* | Forward: GGCCATTGGAAGAACCCTGA | GWHGAOSR008341 |
| *Ge4CL*  *GeCAD* | Reverse: TTCGAAGTTCTGGACGAGGC  Forward: GTGGTTGCACACCGGAGATA  Reverse: TCCTTCATCGGGACAACAGC  Forward: CCACCCATTCGACCCTTTCA  Reverse: ATTGGCAGCACTGAACTCCA | GWHGAOSR011882  GWHGAOSR010533 |
| *GeMYB86* | Forward: AGGTGGTCTCAGATTGCAGC | GWHGBDNU015086 |
|  | Reverse: AGACGTGCTTTACAGGTCCG |  |
| *GeNAC22* | Forward: TTCCGTTGACCATTCCCCTG  Reverse: TTGAGTACAAGGGCGACCAC | GWHTBDNU018183 |
| *GeWRKY35* | Forward: CACAGCTTGGAACAACAGCC | GWHTBDNU020224 |
|  | Reverse: CCATGAGGTCTCCCCAATCG |  |
| *GeWRKY70* | Forward: CACGAGACAGGTGCAGAAGT | GWHTBDNU023036 |
|  | Reverse: TTGCTGATCCCGCTGACTTT |  |
| *Geactin* | Forward: GGGCACAAAAACGTTCCTCC | GWHGAOSR005606 |
|  | Reverse: AAATCCCTCGGCTTGTCCAG |  |
